# Knockdown of Annexin A2 Enhances Radiosensitivity by Increasing G2/M-Phase Arrest, Apoptosis and Activating the p38 MAPK-HSP27 Pathway in Nasopharyngeal Carcinoma

**DOI:** 10.3389/fonc.2022.769544

**Published:** 2022-03-17

**Authors:** Huocong He, Keyu Lin, Changyan Zou, Jianru Pan, Wankai Fu, Yan Zhou, Huamei Lin, Chao Chen, Ying Su

**Affiliations:** ^1^ Laboratory of Radiation Biology and Oncology, Fujian Medical University Cancer Hospital, Fujian Cancer Hospital, Fuzhou, China; ^2^ College of Biological Science and Engineering, Fuzhou University, Fuzhou, China; ^3^ Department of Radiation Oncology, Fujian Medical University Cancer Hospital, Fujian Cancer Hospital, Fuzhou, China; ^4^ Department of Epidemiology, Fujian Medical University Cancer Hospital, Fujian Cancer Hospital, Fuzhou, China; ^5^ Fujian Key Laboratory of Translational Cancer Medicine, Fujian Medical University Cancer Hospital, Fujian Cancer Hospital, Fuzhou, China

**Keywords:** nasopharyngeal carcinoma, annexin A2, radioresistance, cell-cycle arrest, apoptosis

## Abstract

Annexin A2 (ANXA2) has been found to be involved in cancer proliferation, metastasis and prognosis; however, its exact role in nasopharyngeal carcinoma (NPC) radioresistance remains unknown. We found that ANXA2 expression was correlated with prognosis in NPC patients, and longer overall survival in NPC patients with low ANXA2 expression than those with high ANXA2 expression. ANXA2 knockdown increased the radiosensitivity in radioresistant NPC cells, and ANXA2 overexpression decreased the radiosensitivity in NPC cells. Knocking-down ANXA2 expression increased the irradiation-induced apoptosis of radioresistant NPC cells, and ANXA2 overexpression decreased the irradiation-induced apoptosis of NPC cells. ANXA2 knockdown induced G2/M phase arrest in NPC cells post-irradiation, and ANXA2 overexpression abrogated G2/M phase arrest in NPC cells post-irradiation. ANXA2 overexpression resulted in inhibition of the p38 MAPK-HSP27 pathway, while ANXA2 knockdown resulted in activation of the p38 MAPK-HSP27 pathway. In addition, ANXA2 knockdown increased the radiosensitivity of the xenografted tumors in nude mice. Our data demonstrate that knockdown of Annexin A2 enhanced radiosensitivity in NPC by increasing G2/M-phase arrest, apoptosis and activating the p38 MAPK-HSP27 pathway. ANXA2 may be a promising target used to overcome radioresistance in NPC.

## Introduction

Nasopharyngeal carcinoma (NPC) is the most common primary malignancy in the nasopharynx (1). Although NPC is rare worldwide, this malignancy remains highly prevalent in endemic regions, notably in southern China (2, 3). The limitations of anatomic structures and locally infiltrated growth contraindicate surgery for NPC patients, and this malignancy is shown to be insensitive to common chemotherapeutics; therefore, radiotherapy is currently the treatment of choice for NPC (1, 4, 5). Although the 5-year local control rate is reported to achieve 80% to 90% in NPC, there are still some patients experiencing treatment failure (6). Local recurrence and distal metastasis have been identified as the major causes responsible for the death of NPC, which may be attributed to the cellular components resistant to radiation (7, 8). Nevertheless, the mechanisms underlying NPC radioresistance have not been completely demonstrated (9), and there is little knowledge on the biomarkers that may precisely predict the radiosensitivity in NPC until now (10). Therefore, understanding of the molecular mechanisms of NPC radioresistance may provide an opportunity to develop more effective treatments.

Annexin A2 (ANXA2), a member of the annexin family of cytosolic calcium-dependent phospholipid-binding protein, is primarily located in cell membrane, and is also expressed in the cell nucleus (11). Aberrant ANXA2 expression is detected in multiple cancers, and ANXA2 expression is reported to correlate with cancer proliferation, invasion and metastasis, which may be used as biomarker for cancer staging and prognosis prediction (12–15). However, there is little knowledge on the correlation between ANXA2 and radioresistance in NPC until now.

To investigate the mechanisms of NPC radioresistance, we generated a radioresistant NPC CNE2 cell line [CNE2(R743)] by repeated exposure to radiation. Comparative proteomics analysis of radiosensitive CNE2 cells and radioresistant CNE2(R743) cells screened 24 differentially expressed proteins, and the subsequent qPCR assay and Western blotting revealed higher ANXA2 expression in radioresistant CNE2(R743) cells than in radiosensitive CNE2 cells. It is therefore hypothesized that ANXA2 overexpression may contribute to radioresistance in NPC. However, the exact role of ANXA2 in NPC radioresistance and the underlying mechanisms remain unknown. The present study was therefore designed to investigate the molecular mechanisms underlying the contribution of ANXA2 to radioresistance in NPC.

## Materials and Methods

### Human NPC Specimens

A total 100 paraffin-embedded human NPC specimens were sampled from the biopsy specimens of NPC patients that were admitted to Fujian Cancer Hospital (Fuzhou, China) in 2012, and the diagnosis of NPC was confirmed by pathological examinations. NPC was staged according to the American Joint Committee on Cancer (AJCC) TNM staging system (16). The demographic and clinical data were captured from the patients’ medical records.

### Cell Lines and Culture

Human NPC cell line CNE2 was maintained in the Laboratory of Radiation biology and Oncology, Fujian Cancer Hospital (Fuzhou, China), and the radioresistant NPC cell line CNE2(R743) was generated from parental CNE2 cells as previously described (17) and maintained in the Laboratory of Radiation biology and oncology, Fujian Cancer Hospital (Fuzhou, China). Cells were cultured RPMI-1640 medium (Hyclone; Logan, UT, USA) supplemented with 10% fetal bovine serum (FBS; Zhejiang Tianhang Biological Technology Co., Ltd., Deqing, China) at 37°C containing 5% CO_2_.

### Irradiation

All irradiations were delivered using 6 MV X-rays with a linear accelerator (Elekta; Stockholm, Sweden) at a dose-rate of 400 cGy/min and a source surface distance of 100 cm.

### Cell Transfection

SureSilencing shRNA targeting human ANXA2 gene (insert sequence, GTCTGAATTCAAGAGAAAGTA) was linked with the pGeneClip vector (SABiosciences; Frederick, MD, USA) for constructing pGeneClip-ANXA2 shRNA plasmid vector. and the construct was then transfected into CNE2(R743) cells to generate cells stably downexpressing ANXA2 (shR-ANXA2). The control (shR-C) was only transfected with the pGeneClip vector. The efficiency of ANXA2 knockdown was checked using qPCR and Western blotting assays.

To overexpress ANXA2, the full length of the ANXA2 gene was amplified by PCR for constructing pcDNA3.1(+)-ANXA2 plasmid vector (Invitrogen; Thermo Fisher Scientific, Inc., USA) using the designed primers (F: 5′-GGGGTACCATGTCTACTGTTCACGAAATCC-3′; R: 5′-GCTCTAGACTAGTCATCTCCACCACACA-3′), and the construct was then transfected into CNE2 cells to generate cells stably overexpressing ANXA2 (pcD-ANXA2). The control (pcD-C) was only transfected with the pcDNA3.1(+) vector. The ANXA2 gene and protein expression was determined using qPCR and Western blotting assays.

### Clonogenic Survival Assay

The radioresistance levels of the CNE2(R743) cells, shR-C cells, shR-ANXA2 cells, CNE2 cells, pcD-C cells and pcD-ANXA2 cells were measured using clonogenic survival assays following irradiation as previously described (18). Briefly, cells were plated into 6-well plates (Corning, Inc.; Corning, NY, USA) and exposed to irradiation at doses of 0 to 10 Gy: 100 cells/well exposed to 0 Gy irradiation, 200 cells/well exposed to 2 Gy irradiation, 400 cells/well exposed to 4 Gy irradiation, 800 cells/well exposed to 6 Gy irradiation, 1600 cells/well exposed to 8 Gy irradiation and 3200 cells/well exposed to 10 Gy irradiation. Following irradiation, cells were cultured for 14 days and the survival colonies (defined as a colony consists of 50 or more cells) were counted. The survival fraction (SF) was calculated as the number of colonies divided by the number of cells seeded multiplied by the plating efficiency (PE). PE was calculated as the number of colonies for every 100 cells. Sensitivity enhancing ratio (SER) was calculated using the following formula: SER(D_0_)= *D*_0_ in the blank control or negative control group/*D*_0_ in the transfection group, where *D*_0_ indicates the mean lethal dose. All assays were repeated in triplicate.

### Analysis of Apoptosis and Cell Cycle

Apoptosis was detected using flow cytometry with the Annexin V-FITC apoptosis kit (BD Biosciences; San Jose, CA, USA). Cells were exposed to X-ray irradiation at 4 Gy, and cultured for 48 h, and then stained with Annexin V/propidium iodide (PI) for 15 min. The apoptotic fraction was detected on a FACSCanto II flow cytometer (BD Bioscience; San Jose, CA, USA).

Cell cycle was analyzed using flow cytometry. Cells were exposed to 4 Gy X-ray irradiation, cultured for 24 h and then, the cell cycle analysis was performed using flow cytometry with the Cycletest Plus DNA Reagent Kit (BD Bioscience; San Jose, CA, USA) following the manufacturer’s instructions. The flow cytometry data were processed using the software ModFit LT version 3.3. All measurements were repeated in triplicate.

### qPCR Assay

Total RNA was extracted with the RNeasy Mini Kit (Qiagen; Valencia, CA, USA), and the quality and concentration of RNA was determined using gel electrophoresis and ND1000 spectrophotometer (Thermo Fisher Scientific; San Jose, CA, USA). Total RNA was transcribed reversely into cDNA using the RevertAid First Strand cDNA Synthesis Kit (Thermo Fisher Scientific; San Jose, CA, USA) according to the manufacturer’s instructions, and qPCR assay was performed with the LightCycler 480 SYBR Green I Master Kit (Roche Diagnostics, Basel, Switzerland) on a LightCycler 480 II Real-Time PCR System (Roche Diagnostics, Basel, Switzerland) according to the manufacturer’s protocol, using the primer (ANXA2, F: 5′-ACCTGGAGACGGTGATTT-3′, R: 5′-TGCTCTTCTACCCTTTGC-3′) designed by the Sangon Biotech (Shanghai) Co., Ltd. (Shanghai, China),while β-actin (F: 5′-GGAAATCGTGCGTGAC-3′, R: 5′-ATGCCCAGGAAGGAA-3′) served as an internal control. The relative quantity was calculated using the 2^-ΔΔCt^ method. All assays were repeated in triplicate.

### Western Blotting Analysis

Total protein from cultured cells was extracted using the RIPA Lysis and Extraction Buffer (Cell Signaling Technology, Inc.; Danvers, MA, USA), separated by SDS-polyacrylamide gel electrophoresis (SDS-PAGE). The blots were transferred to a nitrocellulose blotting membrane (GE Healthcare Life Science; Freiburg, Germany) in a wet transfer chamber (Bio-Rad; Richmond, CA, USA) at 100 V for 60 min. After blocking with 3% of bovine serum albumin (BSA; Amresco, Solon, OH, USA) for 12 h at 4°C, the membrane was incubated with the specific anti-ANXA2 monoclonal antibody (Cell Signaling Technology, Inc.; Danvers, MA, USA), anti-β-actin monoclonal antibody (Cell Signaling Technology, Inc.; Danvers, MA, USA), anti-Cyclin B1 monoclonal antibody (Cell Signaling Technology, Inc.; Danvers, MA, USA), anti-CDK1 monoclonal antibody (Abcam; Cambridge, MA, USA), anti-Bax polyclonal antibody (Proteintech Group; Rosemont, IL, USA), anti-Bcl-2 polyclonal antibody (Proteintech Group; Rosemont, IL, USA), anti-Vimentin monoclonal antibody (Cell Signaling Technology, Inc.; Danvers, MA, USA), anti-phospho-Vimentin monoclonal antibody (Cell Signaling Technology, Inc.; Danvers, MA, USA), anti-HSP27 monoclonal antibody (Cell Signaling Technology, Inc.; Danvers, MA, USA), anti-phospho-HSP27 monoclonal antibody (Cell Signaling Technology, Inc.; Danvers, MA, USA), anti-p38 MAPK monoclonal antibody (Cell Signaling Technology, Inc.; Danvers, MA, USA) and anti-phospho-p38 MAPK monoclonal antibody (Cell Signaling Technology, Inc.; Danvers, MA, USA) on a rocking platform for 4 h, washed, and then incubated with the goat anti-mouse IgG (H+L) or goat anti-rabbit IgG (H+L) peroxidase-conjugated secondary antibody (Pierce Chemical; Dallas, TX, USA) at room temperature for 3 h, while β-actin served as a loading control. The immunoblot was visualized using the SuperSignal West Femto chemiluminescent substrate (Pierce Chemical; Dallas, TX, USA) in the Image Station 4000MM PRO (Carestream Health, Inc., New Haven, USA).

### Co-Immunoprecipitation (IP) and Matrix-Assisted Laser Desorption/Ionization Time of Flight Mass Spectrometry (MALDI-TOF-MS)

Log-phase CNE2(R743) cells were harvested, washed in cooled PBS, centrifuged, and the supernatant was discarded. The sediment was re-suspended in PBS containing 1 mM PMSF (Thermo Fisher Scientific; San Jose, CA, USA), pieced on ice using ultrasound, centrifuged at 4°C, 12000 r/min for 10 min. The supernatant was collected, and the concentration of total protein was quantified using the BCA assay. Co-IP was performed using the Pierce™ Co-Immunoprecipitation Kit (Pierce Chemical; Dallas, TX, USA) following the manufacturer’s instructions, and the ANXA2-interacting protein enriched by Co-IP was separated using SDS-PAGE. The bands were identified using MALDI-TOF-MS, and MS analysis of peptide was performed on an AB SCIEX MALDI TOF-MS 4700 Analyzer (AB SCIEX, Foster City, CA). The proteins were characterized in the NCBInr database using the online Mascot server, and the ANXA2-interacting proteins were analyzed in the STRING database (http://string-db.org/) as previously described (19).

### Animals

6 weeks old male BALB/c nude mice (SPF grade, weighing 18 ~ 22 g), a total of 36, were purchased from Shanghai Slack Laboratory Animal Co., Ltd. (Shanghai, China). The mice were fed in the Laboratory Animal Center of Fuzhou General Hospital of Nanjing Military Command (Fuzhou, China), and the feeding was accord with the standard for nutritional feed of medical experimental animals. The nude mice were randomly divided into 3 groups (12 mice/group) and inoculated into the following three different tumor cells (1 × 10^6^ cells/100 μL/site): CNE2(R743), shR-C and shR-ANXA2. After the tumor formation, the nude mice were further randomly divided into groups with or without irradiation treatment, that is, a total of 6 groups (6 mice/group): 0 Gy CNE2(R743) group, 10 Gy CNE2(R743) group; 0 Gy shR-C group, 10 Gy shR-C group; 0 Gy shR-ANXA2 group and 10 Gy shR-ANXA2 group. Then, the nude mice were anesthetized by intraperitoneal injection of pentobarbital sodium(dose was 40 mg/kg body weight), and groups of receiving treatment were irradiated with a single 10 Gy dose. Tumor size was measured with a caliper, and tumor volumes (TVs) were calculated using the formula TV = LD^2^/2 (where L was the longest diameter and D was the shortest diameter). After 3 weeks, the mice were sacrificed by cervical dislocation and the tumor tissues were harvested.

### Statistical Analysis

All measurement data were tested for normality using Kolmogorov-Smirnov test, and differences of means between groups were compared with Student’s *t*-test, while the differences among groups were compared with one-way analysis of variance (ANOVA). Comparisons of the count data were done with chi-square test or Fisher’s exact test. The overall survival (OS) and Locoregional recurrence-free survival(LRRFS) were estimated with the Kaplan-Meier method, and the OS and LRRFS were compared with log-rank test. The associations of ANXA2 expression with the clinicopathological variables were examined with partial correlation analysis. All statistical analyses were performed using the statistical software SPSS version 18.0 (SPSS, Inc.; Chicago, IL, USA), and a *P* value of < 0.05 was considered statistically significant.

## Results

### ANXA2 Overexpression Predicts Poor Survival in NPC Patients

**Figure 1:**
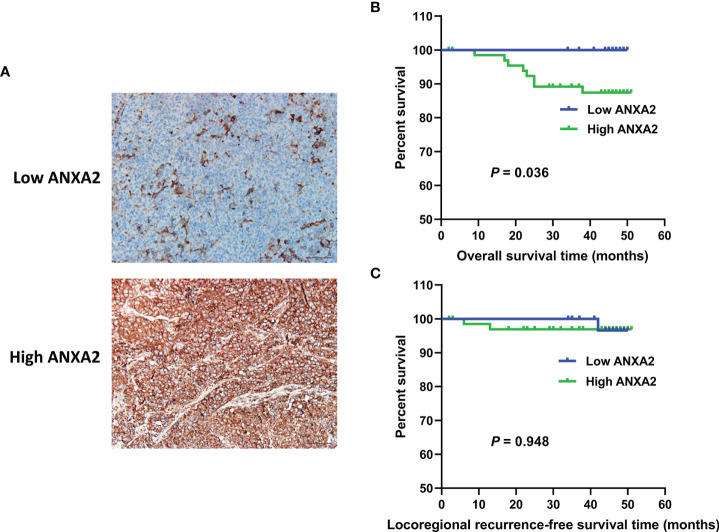
ANXA2 expression in NPC specimens and its association with overall survival and Locoregional recurrence-free survival in NPC patients. **(A)** immunohistochemical analysis of ANXA2 expression in NPC specimens, scale bar = 50 μm; **(B, C)** Kaplan-Meier survival curves of NPC patients with high (*n* = 67) and low (*n* = 33) ANXA2 expression, and the comparison of overall survival **(B)** and Locoregional recurrence-free survival **(C)** were done using a log-rank test.

To examine the correlation between ANXA2 expression and NPC prognosis, immunohistochemistry (IHC) was performed to determine ANXA2 expression in human NPC specimens, and 67% specimens were positive for ANXA2. Partial correlation analysis revealed that the ANXA2 expression was not associated with the gender (*P* = 0.871), age (*P* = 0.656), pathologic type (*P* = 0.699), clinical stage (*P* = 0.126), tumor status (*P* = 0.663), lymph node status (*P* = 0.151), distal metastasis status (*P* = 0.330) and recurrence (*P* = 0.704), but correlated with prognosis in NPC patients (*P* = 0.036) (**Table 1**). In addition, Kaplan-Meier survival analysis revealed longer OS in NPC patients with low ANXA2 expression than in those with high ANXA2 expression (*P* = 0.036) (**Figure 1**). However, there was no significant difference in LRRFS between ANXA2 high and low expression groups (*P* = 0.948) (**Figure 1**). These data indicate that high ANXA2 expression predicts poor prognosis in patients with NPC.

**Table 1:** Associations of ANXA2 expression with clinicopathological characteristics in nasopharyngeal carcinoma patients.

Clinicopathological characteristics		No. cases		*P*
		High ANXA2 expression (*n* = 67)	Low ANXA2 expression (*n* = 33)	
Gender	Male	56	28	0.871
	Female	11	5	
Age (years)	≤ 50	45	20	0.656
	> 50	22	13	
Pathologic type	Undifferentiated carcinoma without cornification	65	33	0.699
	Undifferentiated carcinoma with cornification	2	0	
Clinical stage	I	1	4	0.126
	II	5	2	
	III	30	16	
	IV	31	11	
Tumor status	T1 and T2	26	11	0.663
	T3 and T4	41	22	
Lymph node status	N0	4	5	0.151
	N1–N3	63	28	
Distal metastasis status	M0	67	32	0.330
	M1	0	1	
Recurrence	No	65	32	0.704
	Yes	2	1	
Prognosis	Death	8	0	0.036
	Survival	59	33	

Low ANXA2, low level for ANXA2 expression; High ANXA2, high level for ANXA2 expression.

### ANXA2 Knockdown Increases Radiosensitivity and ANXA2 Overexpression Increases Radioresistance in NPC Cells

**Figure 2:**
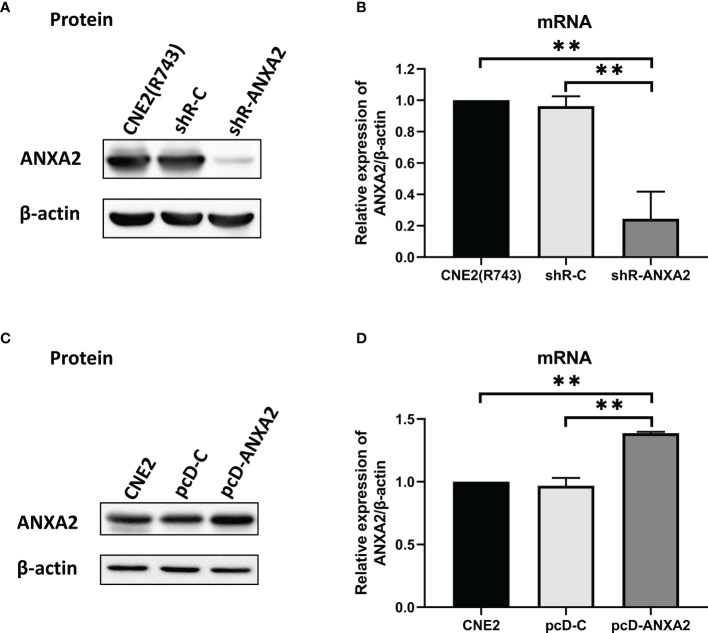
Generation of ANXA2 knockdown and overexpression in NPC cells. **(A)** Western blotting determines the ANXA2 expression in shR-ANXA2, shR-C and CNE2(R743) cells, and β-actin serves as a loading control; **(B)** qPCR assay quantifies the relative ANXA2 expression in shR-ANXA2, shR-C and CNE2(R743) cells; **(C)** Western blotting determines the ANXA2 expression in pcD-ANXA2, pcD-C and CNE2 cells, and β-actin serves as a loading control; **(D)** qPCR assay quantifies the relative ANXA2 expression in pcD-ANXA2, pcD-C and CNE2 cells. All values are presented as mean ± SD, n = 3. **P < 0.01.

To examine the exact role of ANXA2 in NPC radioresistance, we generated NPC cell lines with stable ANXA2 knockdown and overexpression. qPCR and Western blotting assays showed lower ANXA2 expression in shR-ANXA2 cells than in shR-C cells and CNE2(R743) cells (*P* < 0.05), while no significant difference was detected in the ANXA2 expression between shR-C cells and CNE2(R743) cells (*P* > 0.05) (**Figures 2A, B**), indicating the successful generation of ANXA2 knockdown in shR-ANXA2 cells. In addition, higher ANXA2 expression was detected in pcD-ANXA2 cells than in pcD-C and CNE2 cells (*P* < 0.05), while no significant difference was detected in the ANXA2 expression between pcD-C and CNE2 cells (*P* > 0.05) (**Figures 2C, D**), indicating the successful induction of ANXA2 overexpression in pcD-ANXA2 cells.

**Figure 3:**
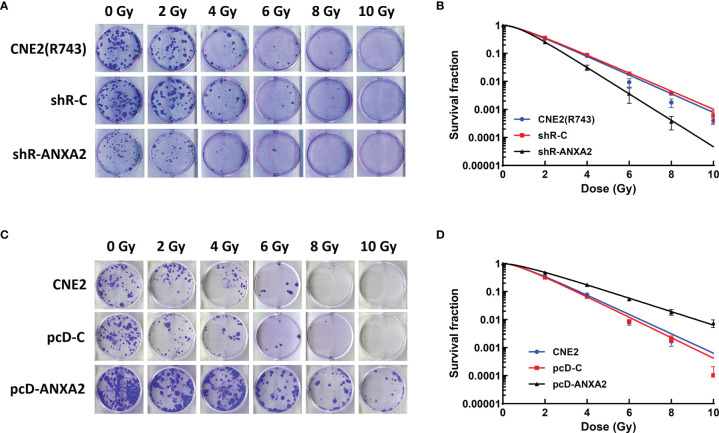
ANXA2 knockdown increases radiosensitivity of NPC cells and ANXA2 overexpression decreases radiosensitivity of NPC cells. **(A)** clonogenic survival assay reveals less cell colonies in shR-ANXA2 cells than in shR-C and CNE2(R743) cells following exposure to X-ray irradiation at doses of 0, 2, 4, 6, 8 and 10 Gy; **(B)** the radiosensitivity is increased in shR-ANXA2 cells than in shR-C and CNE2(R743) cells following exposure to X-ray irradiation at doses of 0, 2, 4, 6, 8 and 10 Gy; **(C)** clonogenic survival assay shows more cell colonies in pcD-ANXA2 cells than in pcD-C and CNE2 cells following exposure to X-ray irradiation at doses of 0, 2, 4, 6, 8 and 10 Gy; **(D)** a decrease is seen in the radiosensitivity in pcD-ANXA2 cells relative to pcD-C and CNE2 cells. All results are given as mean ± SD obtained from three independent experiments.

To assess the association of ANXA2 knockdown with the radiosensitivity in NPC cells, the shR-ANXA2 cells were exposed to X-ray irradiation at doses of 0 to 10 Gy, while shR-C and CNE2(R743) cells served as controls. Clonogenic survival assay revealed less cell colonies in shR-ANXA2 cells than in shR-C and CNE2(R743) cells (**Figure 3A**), and the radiosensitivity was increased in shR-ANXA2 cells than in shR-C and CNE2(R743) cells (**Figure 3B**). Additionally, the SER(D_0_) was 1.41- and 1.47-fold changes in shR-ANXA2 cells from CNE2(R743) and shR-C cells respectively, and the D_0_ was 0.92 in shR-ANXA2 cells, 1.3 in CNE2(R743) cells, and 1.35 in shR-C cells, respectively. These data demonstrate that knockdown of ANXA2 expression increases the radiosensitivity in radioresistant NPC CNE2(R743) cells.

To detect the association of ANXA2 overexpression with the radioresistance in NPC cells, the pcD-ANXA2 and pcD-C and CNE2 cells were exposed to irradiation at doses of 0 to 10 Gy. We found more cell colonies in pcD-ANXA2 cells than in pcD-C and CNE2 cells (**Figure 3C**), and a decrease was seen in the radiosensitivity in pcD-ANXA2 cells relative to pcD-C- and CNE2 cells (**Figure 3D**). Moreover, the SER(D_0_) were 0.65- and 0.61-fold changes in pcD-ANXA2 cells from CNE2 and pcD-C cells, and the D_0_ was 1.94 in pcD-ANXA2 cells, 1.26 in CEN2 cells, and 1.19 in pcD-C cells, respectively. Our findings indicate that overexpression of ANXA2 decreases the radiosensitivity in NPC CNE2 cells.

### ANXA2 Knockdown Increases Irradiation-Induced Apoptosis and ANXA2 Overexpression Decreases Irradiation-Induced Apoptosis in NPC Cells

**Figure 4:**
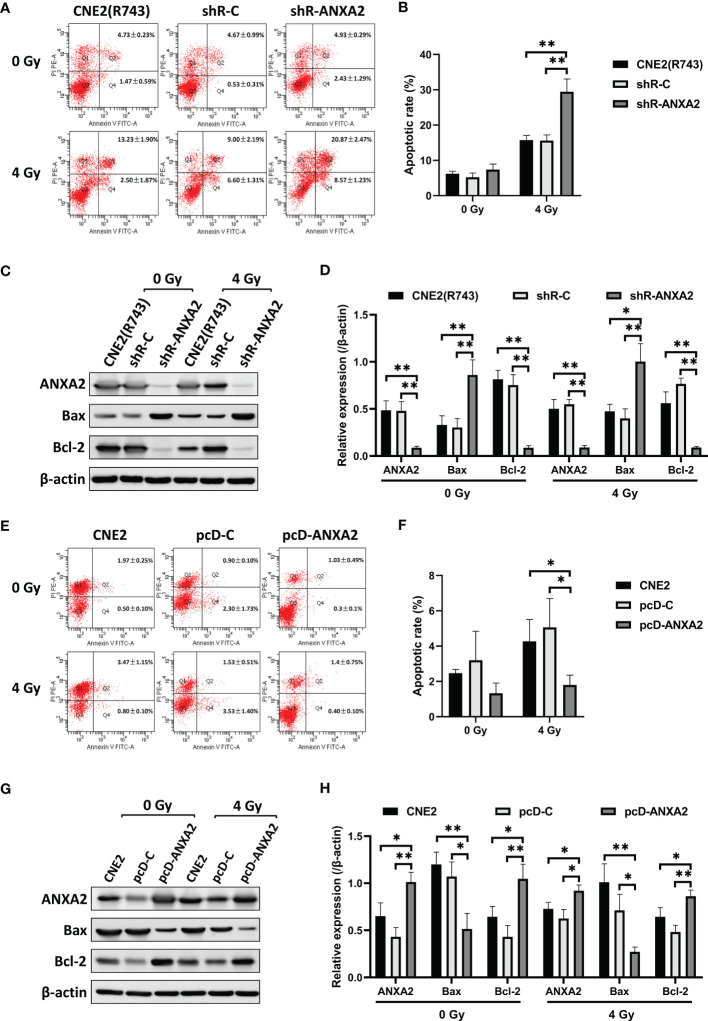
ANXA2 knockdown increases irradiation-induced apoptosis and ANXA2 overexpression decreases irradiation-induced apoptosis in NPC cells. **(A)** flow cytometric analysis detects the apoptosis of shR-ANXA2, shR-C and CNE2(R743) cells 48 h post-exposure to irradiation at doses of 0 and 4 Gy; **(B)** a higher apoptotic rate is measured in shR-ANXA2 cells than in shRNA-C and CNE2(R743) cells 48 h post-exposure to irradiation at a dose of 4 Gy; **(C)** Western blotting determines the expression of apoptosis-related proteins in shR-ANXA2, shR-C and CNE2(R743) cells 48 h post-exposure to irradiation at doses of 0 and 4 Gy, and β-actin serves as a loading control; **(D)** higher Bax expression and lower Bcl-2 expression is determined in shR-ANXA2 cells than in shR-C and CNE2(R743) cells 48 h after 4 Gy irradiation; **(E)** flow cytometric analysis detects the apoptosis of pcD-ANXA2, pcD-C and CNE2 cells 48 h post-exposure to irradiation at doses of 0 and 4 Gy; **(F)** a lower apoptotic rate is detected in pcD-ANXA2 cells than in pcD-C and CNE2 cells; **(G)** Western blotting determines the expression of apoptosis-related proteins in pcD-ANXA2, pcD-C and CNE2 cells 48 h post-exposure to irradiation at doses of 0 and 4 Gy, and β-actin serves as a loading control; **(H)** lower Bax and higher Bcl-2 expression is determined in pcD-ANXA2 cells than in pcD-C and CNE2 cells. Data are presented as mean ± SD of three experiments. *P < 0.05, **P < 0.01.

Flow cytometry measured a higher apoptotic rate of shR-ANXA2 cells in relative to shR-C and CNE2(R743) cells 48 h post-exposure to irradiation at a dose of 4 Gy (*P* < 0.01) (**Figures 4A, B**). Then, we determined the expression of apoptosis-related proteins, including Bax and Bcl-2, and Western blotting showed higher Bax expression and lower Bcl-2 expression in shR-ANXA2 cells than in shR-C and CNE2(R743) cells 48 h following exposure to irradiation at a dose of 4 Gy (*P* < 0.05) (**Figures 4C, D**). These data demonstrate that shRNA-induced ANXA2 knockdown increases the irradiation-induced apoptosis of the radioresistant NPC CNE2(R743) cells.

We further assessed the effect of ANXA2 overexpression on irradiation-induced apoptosis of CNE2 cells. Flow cytometry detected a lower apoptotic rate of pcD-ANXA2 cells in relative to pcD-C and CNE2 cells (*P* < 0.05) (**Figures 4E, F**), and Western blotting determined lower Bax and higher Bcl-2 expression in pcD-ANXA2 cells than in pcD-C and CNE2 cells (*P* < 0.05) (**Figures 4G, H**). The results indicate that ANXA2 overexpression decreases the irradiation-induced apoptosis of NPC cells.

### ANXA2 Knockdown Induces and ANXA2 Overexpression Abrogates G2/M Cell Cycle Arrest in NPC Cells Following Irradiation

**Figure 5:**
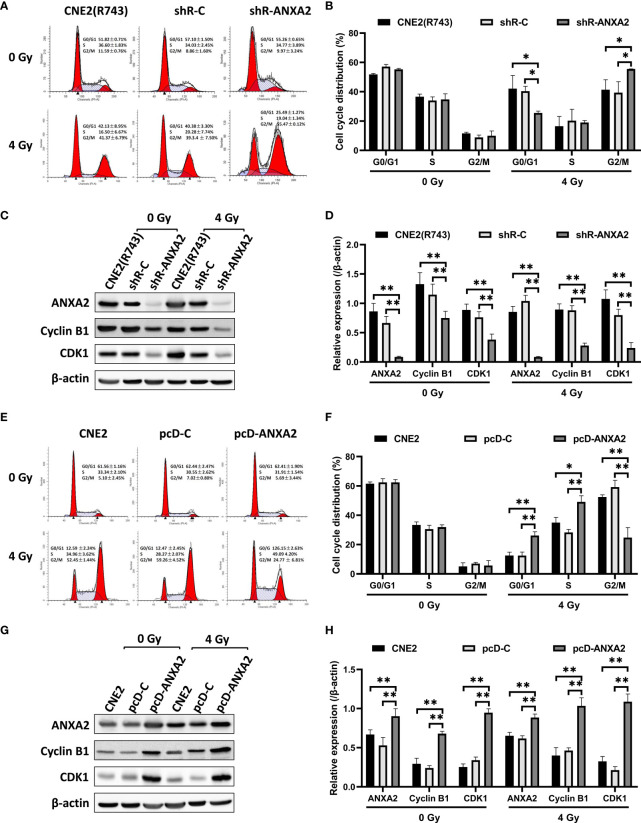
ANXA2 knockdown induces and ANXA2 overexpression abrogates G2/M cell cycle arrest in NPC cells following irradiation. **(A)** flow cytometric analysis detects the cell cycle distribution of shR-ANXA2, shR-C and CNE2(R743) cells 24 h post-exposure to irradiation at doses of 0 and 4 Gy; **(B)** the percentage of shR-ANXA2 cells with G2/M cell-cycle arrest is significantly higher than those of shR-C and CNE2(R743) cells 24 h following exposure to irradiation at 4 Gy, no significant difference is seen in the percentage of shR-ANXA2, shR-C and CNE2(R743) cells with G2/M cell-cycle arrest without irradiation; **(C)** Western blotting determines the expression of cell cycle-related proteins in shR-ANXA2, shR-C and CNE2(R743) cells 24 h post-exposure to irradiation at doses of 0 and 4 Gy, and β-actin serves as a loading control; **(D)** lower Cyclin B1 and CDK1 expression is determined in shR-ANXA2 cells than in shR-C and CNE2(R743) cells; **(E)** flow cytometric analysis detects the cell cycle distribution of pcD-ANXA2, pcD-C and CNE2 cells 24 h post-exposure to irradiation at doses of 0 and 4 Gy; **(F)** a lower proportion of the pcD-ANXA2 cells with G2/M cell-cycle arrest is measured in relative to pcD-C and CNE2 cells 24 h following exposure to irradiation at 4 Gy, and no significant difference was detected in the proportion of pcD-ANXA2, pcD-C and CNE2 cells with G2/M cell-cycle arrest without irradiation; **(G)** Western blotting determines the expression of cell cycle-related proteins in pcD-ANXA2, pcD-C and CNE2 cells 24 h post-exposure to irradiation at doses of 0 and 4 Gy, and β-actin serves as a loading control; **(H)** higher Cyclin B1 and CDK1 expression is detected in pcD-ANXA2 cells than in pcD-C and CNE2 cells. Data are presented as mean ± SD of three experiments. *P < 0.05, **P < 0.01.

As shown above, knockdown of ANXA2 expression increases the sensitivity of NPC cells to irradiation. Since the therapeutic effect of irradiation is affected by the cell-cycle phase (20), we evaluated the role of ANXA2 knockdown in the alteration of the cell-cycle phase caused by irradiation. After 24 h following exposure to irradiation at 4 Gy, the percentage of shR-ANXA2 cells with G2/M cell-cycle arrest was significantly higher than those of shR-C and CNE2(R743) cells (*P* < 0.05); however, no significant difference was seen in the percentage of shR-ANXA2, shR-C and CNE2(R743) cells with G2/M cell-cycle arrest without irradiation (*P* > 0.05) (**Figures 5A, B**). Then, we determined the expression of cell cycle-related proteins Cyclin B1 and CDK1 in CNE2(R743) cells, and Western blotting detected lower Cyclin B1 and CDK1 expression in shR-ANXA2 cells than in shR-C and CNE2(R743) cells (*P* < 0.01) (**Figures 5C, D**). These data indicate that ANXA2 knockdown induces G2/M cell-cycle arrest in NPC cells post-irradiation.

We further examined the role of ANXA2 overexpression in the changes of cell-cycle phase induced by irradiation. Flow cytometry showed a lower proportion of the pcD-ANXA2 cells with G2/M cell-cycle arrest in relative to pcD-C and CNE2 cells (*P* < 0.01) after 24 h following exposure to irradiation at 4 Gy; however, no significant difference was detected in the proportion of pcD-ANXA2, pcD-C and CNE2 cells with G2/M cell-cycle arrest without irradiation (*P* > 0.05) (**Figures 5E, F**). In addition, Western blotting analysis revealed higher Cyclin B1 and CDK1 expression in pcD-ANXA2 cells than in pcD-C and CNE2 cells (*P* < 0.01) (**Figures 5G, H**). Our findings demonstrate that ANXA2 overexpression abrogates G2/M cell-cycle arrest in NPC cells post-irradiation.

### p38 MAPK-HSP27 Pathway Is Activated by ANXA2 Knockdown and Inactivated by ANXA2 Overexpression

**Figure 6:**
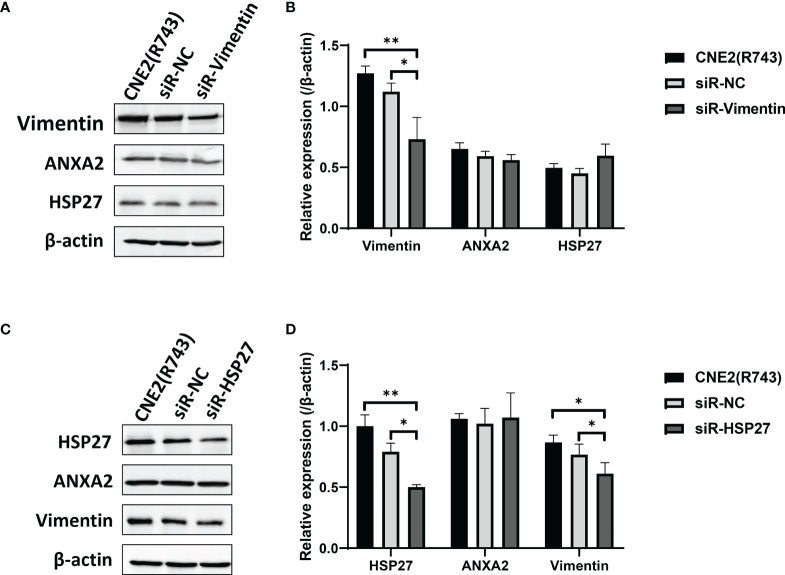
Downregulation of Vimentin and HSP27 expression has no impact on ANXA2 expression. **(A)** Western blotting determines the expression of ANXA2 and ANXA2-interacting proteins HSP27 and Vimentin in siR-Vimentin-, siR-NC- (Negative control siRNA) and non-transfected radioresistant CNE2(R743) cells, and β-actin serves as a loading control; **(B)** densitometric analysis determines lower Vimentin expression in siR-Vimentin-transfected CNE2(R743) cells than in siR-NC- and non-transfected CNE2(R743) cells, and no significant difference was seen in ANXA2 or HSP27 expression among the siR-Vimentin-, siR-NC- and non-transfected CNE2(R743) cells; **(C)** Western blotting determines the expression of ANXA2 and ANXA2-interacting proteins HSP27 and Vimentin in siR-HSP27-, siR-NC- and non-transfected radioresistant CNE2(R743) cells, and β-actin serves as a loading control; **(D)** densitometric analysis determines lower Vimentin and HSP27 expression in siR-HSP27-transfected CNE2(R743) cells than in siR-NC- and non-transfected CNE2(R743) cells, and no significant difference was seen in ANXA2 expression among the siR-HSP27-, siR-NC- and non-transfected CNE2(R743) cells. All values are presented as mean ± SD, n = 3. *P < 0.05, **P < 0.01.

We next investigated the possible mechanisms underlying ANXA2-mediated radiosensitivity in NPC cells. Firstly, Co-IP and MALDI-TOF-MS were employed to screen 5 ANXA2-interacting proteins in radioresistant CNE2(R743) cells, and HSP27 and Vimentin were found to interact with ANXA2 by using the STRING database. However, siRNA-induced Vimentin knockdown showed no effect on ANXA2 or HSP27 expression (**Figures 6A, B**), while HSP27 knockdown downregulated Vimentin expression but did not affect ANXA2 expression (**Figures 6C, D**). It is therefore hypothesized that ANXA2 may be an upstream signaling molecule of the p38MAPK-HSP27 pathway.

**Figure 7:**
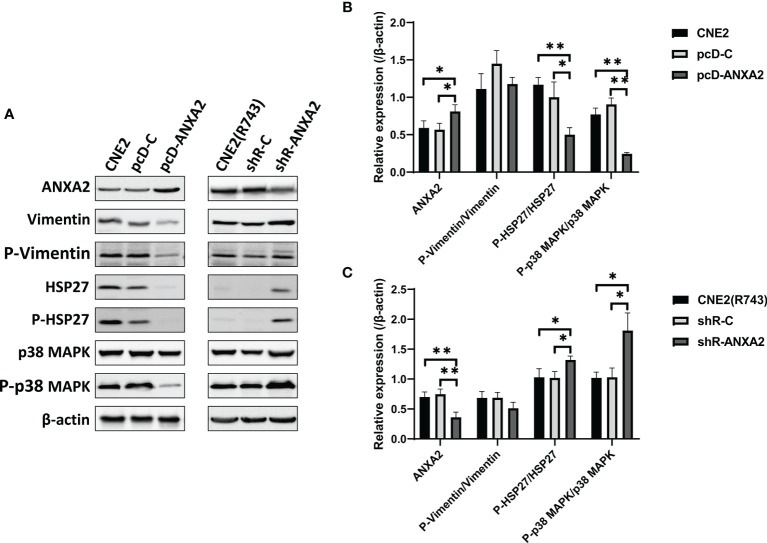
p38 MAPK-HSP27 pathway is activated by ANXA2 knockdown and inactivated by ANXA2 overexpression. **(A)** Western blotting determines the expression of the p38 MAPK-HSP27 pathway-associated proteins in NPC cells 48 h post-exposure to X-ray irradiation at a dose of 4 Gy, and β-actin serves as a loading control; **(B)** lower phospho-HSP27/HSP27 and phospho-p38MAPK/p38MAPK expression are determined in pcD-ANXA2 cells than in pcD-C and CNE2 cells; **(C)** higher phospho-HSP27/HSP27 and phospho-p38 MAPK/p38 MAPK expression are detected in shR-ANXA2 cells than in shR-C and CNE2(R743) cells. All values are presented as mean ± SD, n = 3. *P < 0.05, **P < 0.01. P-Vimentin, phospho-Vimentin; P-HSP27, phospho-HSP27; P-p38MAPK, phospho-p38MAPK.

To test the hypothesis, ANXA2-overexpressing CNE2 cells were exposed to X-ray irradiation at 4 Gy, cultured for 48 h and then, the expression of key signaling molecules associated with the p38MAPK-HSP27 pathway was detected. Western blotting revealed lower phospho-HSP27/HSP27, and phospho-p38MAPK/p38MAPK expression in pcD-ANXA2 cells than in pcD-C and CNE2 cells (*P* < 0.01), (**Figures 7A, B**), indicating that ANXA2 overexpression results in inhibition of the p38MAPK-HSP27 signaling pathway. To further examine the role of ANXA2 in the p38MAPK-HSP27 signaling pathway, we determined the expression of key signaling molecules associated with the p38MAPK-HSP27 pathway in ANXA2-knockdown radioresistant CNE2(R743) cells after 48 h following exposure to irradiation at 4 Gy. Western blotting showed upregulation of the phospho-HSP27/HSP27 and phospho-p38MAPK/p38MAPK expression in shR-ANXA2 cells as compared to that in shR-C and CNE2(R743) cells (*P*<0.05) (**Figures 7A, C**), which demonstrates that ANXA2 knockdown results in activation of the p38MAPK-HSP27 signaling pathway.

### Knockdown of ANXA2 Expression Increases the Radiosensitivity of Xenografted Tumors in a Nude Mice Model of NPC

**Figure 8:**
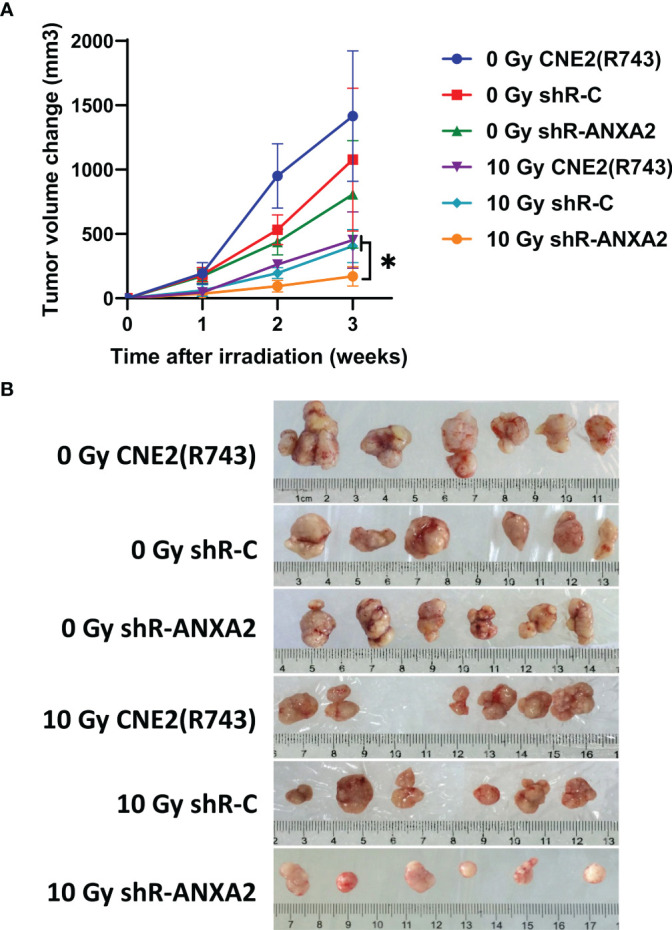
Knockdown of ANXA2 expression increases the radiosensitivity of xenografted tumors in a nude mice model of NPC. **(A)** the volumes of the xenografted tumors from shR-ANXA2, shR-C and CNE2(R743) 1, 2 and 3 weeks post-irradiation at doses of 0 and 10 Gy; **(B)** the size of the xenografted tumors from shR-ANXA2, shR-C and CNE2(R743) 3 weeks post-irradiation at doses of 0 and 10 Gy. Values are presented as mean ± SD. *P < 0.05.

The tumor xenografted nude mice were exposed to X-ray irradiation at 0 and 10 Gy, and the volume of xenografted tumors was measured 1, 2 and 3 weeks post-irradiation. The growth of xenografted tumors was faster in mice without irradiation than in those exposed to radiation. Without irradiation, the volumes of xenografted tumors from shR-ANXA2, shR-C and CNE2(R743) cells showed no significant differences 1, 2 and 3 weeks (*P* > 0.05). Following exposure to X-ray irradiation at 10 Gy, the slowest growth was seen in the xenografted tumors from shR-ANXA2 cells, and the volume of xenografted tumors from shR-ANXA2 cells was smaller than those from shR-C and CNE2(R743) cells at 3 weeks (*P* < 0.05) (**Figures 8A, B**). These data demonstrate that ANXA2 knockdown increases the radiosensitivity of the xenografted tumors in nude mice.

## Discussion

In this study, IHC detected high ANXA2 expression in human NPC specimens, and partial correlation analysis revealed no associations of ANXA2 expression with the gender, age, pathologic type, clinical stage, tumor status, lymph node status, distal metastasis status and recurrence; however, low ANXA2 expression was associated with improved OS in NPC patients. These data suggest that ANXA2 overexpression may be a predictor of poor prognosis in NPC patients. Previous studies have shown that ANXA2 overexpression is strongly associated with the progression of multiple malignancies (21–24), and promotes the metastatic potential of endometrial cancer (25, 26), kidney cancer (27, 28) and colorectal cancer (29, 30). A recent meta-analysis to evaluate the prognostic value of ANXA2 overexpression in malignant tumors revealed that ANXA2 overexpression correlated with poor outcomes in patients with malignant tumors (31). Taken together, these data demonstrate that aberrant ANXA2 expression is an important prognostic factor in multiple cancers, and correlates with malignancy progression.

To assess the effect of ANXA2 overexpression on NPC radiosensitivity, ANXA2 expression was knocked down in radioresistant CNE2(R743) cells, and the ANXA2-knockdown radioresistant CNE2(R743) cells were xenografted into nude mice to establish a murine model of NPC. *In vivo* study showed that the xenografted tumors from shR-ANXA2-transfected CNE2(R743) cells were more sensitive to X-ray irradiation than those from shR-C and CNE2(R743) cells, which was consistent with the results of the clonogenic survival assay. In addition, *in vitro* cellular assay revealed that ANXA2 knockdown increased the radiosensitivity and ANXA2 overexpression decreased the radiosensitivity in NPC cells. Our showed that low ANXA2 expression may predict a longer OS in NPC patients and animal experiments indicated that knockdown of ANXA2 expression increased the radiosensitivity in NPC xenografts. However, the molecular mechanism underlying the link between aberrant ANXA2 expression and NPC radioresistance remains unclear until now.

Cell-cycle arrest is one of the most common causes for the radiosensitizing effect (32, 33). Exposure to X-ray or γ-ray irradiation causes a cell-division delay including G1 or G2 cell-cycle arrest or S phase delay, and cells at the G2/M phase are most sensitive to ionizing radiation (34–36). In this study, shRNA-induced knockdown of ANXA2 was found to induce G2/M cell-cycle arrest and ANXA2 overexpression abrogated G2/M arrest in NPC cells post-exposure to X-ray irradiation. Cyclin B1 is a cell cycle-associated protein that accumulates and forms a kinase complex with CDK1 during the G2/M phase (37), and G2/M arrest has been associated with an inhibition or a delay in the activation of CDK1 in association with their specific regulatory Cyclin B1 proteins (38). Thus, we determined the expression of Cyclin B1 and CDK1 proteins, and Western blotting analysis revealed that ANXA2 knockdown caused a reduction in the Cyclin B1 and CDK1 expression, while ANXA2 overexpression resulted in elevated Cyclin B1 and CDK1 expression in NPC cells exposed to X-ray irradiation. Accumulated evidence suggests that a lot of radiosensitizing compounds function as anti-cancer agents *via* the induction of the G2/M cell-cycle arrest through downregulating Cyclin B and CDK1 expression (39, 40), and fenofibrate was reported to induce G2/M cell-cycle arrest through reducing the activity of the CDK1/Cyclin B1 kinase complex, thereby increasing the radiosensitivity (41).Taken together, it is therefore considered that ANXA2 is involved in the mediation of the G2/M cell-cycle arrest, thereby affecting the efficacy of radiotherapy.

It has been shown that the response of cancer cells to ionizing radiation is closely associated with cell cycle changes and DNA damage repair following irradiation (42, 43), and if DNA damage is not repaired, irradiation will promote the apoptosis (44). Irradiation-induced apoptosis is a degradative and progressive process, and the degradative process is initiated in the target nucleus, ultimately resulting in the quantitative conversion of the target genome into small DNA fragments (45). However, the correlation between ionizing radiation-induced apoptosis and radiosensitivity remains controversial (46, 47). It was reported that the apoptosis of cancer cells had associations with radiosensitivity, while there was also some evidence demonstrating no correlation (47, 48). Previous studies have demonstrated that the downregulation of ANXA2 expression suppresses the efficiency of DNA damage repair following exposure to γ-ray and ultraviolet-B (UVB) irradiation, thereby resulting in cell death (9, 49). In this study, our findings showed that ANXA2 knockdown increased irradiation-induced apoptosis and ANXA2 overexpression decreased irradiation-induced apoptosis in NPC cells. The pro- and anti-apoptotic proteins of the Bcl-2 family may trigger and suppress the apoptosis because of the formation of heterodimers among these proteins, and the heterodimerization results in mutual neutralization of the bound pro-and anti-apoptotic proteins (50). Therefore, the balance between the expression levels of apoptosis-related proteins (Bcl-2 and Bax) is critical for cell survival or apoptosis (51). Therefore, we then determined the expression levels of apoptosis-related proteins in NPC cells, and Western blotting revealed that ANXA2 knockdown caused an increase in the pro-apoptotic Bax protein expression, a reduction in the anti-apoptotic Bcl-2 protein expression and a rise in the Bax/Bcl-2 ratio, while ANXA2 overexpression resulted in a reduction in Bax protein expression, an increase in Bcl-2 expression and a decrease in the Bax/Bcl-2 ratio, which was in agreement with previous findings (52).

To investigate the mechanisms underlying the effect of aberrant ANXA2 expression on NPC radiosensitivity, MALDI-TOF-MS and Co-IP were employed to screen the proteins interacting with ANXA2, and HSP27 and Vimentin were identified to interact with ANXA2. However, knockdown of Vimentin or HSP27 expression showed no effects on ANXA2 expression. It is therefore assumed that HSP27 and Vimentin may be not the upstream target molecules of ANXA2. As a major signaling molecule in the p38 MAPK pathway, HSP27 and p38 MAPK jointly constitutes the p38 MAPK-HSP27 pathway (53). p38 MAPK is activated by a variety of cellular stresses including osmotic shock, inflammatory cytokines, lipopolysaccharide (LPS), UV light, and growth factors (54, 55). Activated p38 MAPK has been shown to phosphorylate and activate MAPKAPK-2, and MAPKAPK-2 activates HSP27 (53). In this study, ANXA2 knockdown was found to upregulate phospho-HSP27/HSP27 and phospho-p38 MAPK/p38 MAPK protein expression, indicating the activation of the p38 MAPK-HSP27 signaling pathway. Taken together, it is hypothesized that shRNA-induced knockdown of ANXA2 expression may increase NPC radiosensitivity *via* the activation of the p38 MAPK-HSP27 signaling pathway.

In addition, our data showed that ANXA2 overexpression resulted in downregulation of phospho-p38 MAPK/p38 MAPK and phospho-HSP27/HSP27 protein expression, indicating the suppression of the p38 MAPK-HSP27 signaling pathway, which results in NPC radioresistance. The p38 MAPK-HSP27 signaling pathway is reported to strongly correlate with reactive oxygen species (ROS), and is associated with apoptosis and radiosensitivity (56). p38 MAPK is a downstream signaling molecule of apoptosis signal-regulated kinase 1 (ASK 1), which is activated by ROS, and excessive ROS triggers DNA damage and activates p53 and p38 MAPK, resulting in an increase in radiosensitivity in human cancer cells (57, 58). Our findings suggest that that ANXA2 knockdown may increase the radiosensitivity in NPC cells exposed to X-ray irradiation through activating the p38 MAPK-HSP27 signaling pathway, upregulating Bax expression and downregulating Bcl-2 expression, and ANXA2 overexpression may decrease the radiosensitivity in NPC cells exposed to X-ray radiation through inhibiting the p38 MAPK-HSP27 signaling pathway, downregulating Bax expression and upregulating Bcl-2 expression. The p38 MAPK pathway has been shown to be closely associated with radiosensitivity in multiple cancers (59, 60). Further studies to examine the effects of aberrant ANXA2 expression induced activation or suppression of the p38 MAPK-HSP27 signaling pathway on NPC radiosensitivity are required.

## Conclusions

In summary, the results of the present study demonstrate that high ANXA2 expression predicts a shorter OS than low ANXA2 expression in NPC patients following radiotherapy, and ANXA2 knockdown reduces the growth of NPC xenografts in nude mice after irradiation. In addition, ANXA2 knockdown causes G2/M cell-cycle arrest, an increase in the apoptotic rate, a reduction in the formation rate of cell colonies and activation of the p38 MAPK-HSP27 pathway in NPC cells post-exposure to X-ray irradiation, and ANXA2 overexpression results in abrogation of the G2/M cell-cycle arrest, a reduction in the apoptotic rate, an increase in the formation rate of cell colonies and suppression of the p38 MAPK-HSP27 pathway in NPC cells post-exposure to X-ray radiation. It is there considered that ANXA2 may be a promising target used to overcome radioresistance in NPC.

## Data Availability Statement

The raw data supporting the conclusions of this article will be made available by the authors, without undue reservation.

## Ethics Statement

The studies involving human participants were reviewed and approved by the Ethical Review Committee of Fujian Cancer Hospital (permission no. FJZLYY-2012-0078). Written informed consent to participate in this study was provided by the participants. The animal study was reviewed and approved by the Animal Ethics Committee of Fuzhou General Hospital of Nanjing Military Command (Fuzhou, China) (permission no. IACUC-2016-30), and all animal experiments were done according to the international and national guidelines on the breeding, care, and management of laboratory animals.

## Author Contributions

HH, KL, CZ, and YS conceived and designed the experiments. HH, JP, and YZ performed the data analyses and interpretation. HH, KL, CZ, WF, HL, and CC carried out the experiments. HH and YS contributed to manuscript writing. All authors read and approved the final manuscript.

## Funding

This study was supported by the grants from Fujian Provincial Key Sci & Tech Project (grant no. 2014Y0014), National Key Clinical Specialty Construction Project of China (grant no. 2013-554), the Natural Science Foundation of Fujian Province (grant no. 2014J01299 and 2019J01191) and the National Natural Science Foundation of China (grant no. 81472907 and 81974482). Sponsored by Fujian Provincial Health Technology Project (grant no:2019-CXB-8).

## Conflict of Interest

The authors declare that the research was conducted in the absence of any commercial or financial relationships that could be construed as a potential conflict of interest.

## Publisher’s Note

All claims expressed in this article are solely those of the authors and do not necessarily represent those of their affiliated organizations, or those of the publisher, the editors and the reviewers. Any product that may be evaluated in this article, or claim that may be made by its manufacturer, is not guaranteed or endorsed by the publisher.
